# Software cost estimation predication using a convolutional neural network and particle swarm optimization algorithm

**DOI:** 10.1038/s41598-024-63025-8

**Published:** 2024-06-07

**Authors:** Moatasem. M. Draz, Osama Emam, Safaa. M. Azzam

**Affiliations:** 1 Software Engineering Department, Faculty of Computers and Information, Kafrelsheikh University, Kafrelsheikh, Egypt; 2 Information Systems Department, Faculty of Computers and Artificial Intelligence, Helwan University, Helwan, Egypt

**Keywords:** Software cost estimation, Software effort estimation, Promise database, SCE, Convolutional neural network, Particle swarm optimization algorithm, PSO, CNN, Computer science, Software

## Abstract

Over the past decades, the software industry has expanded to include all industries. Since stakeholders tend to use it to get their work done, software houses seek to estimate the cost of the software, which includes calculating the effort, time, and resources required. Although many researchers have worked to estimate it, the prediction accuracy results are still inaccurate and unstable. Estimating it requires a lot of effort. Therefore, there is an urgent need for modern techniques that contribute to cost estimation. This paper seeks to present a model based on deep learning and machine learning techniques by combining convolutional neural networks (CNN) and the particle swarm algorithm (PSO) in the context of time series forecasting, which enables feature extraction and automatic tuning of hyperparameters, which reduces the manual effort of selecting parameters and contributes to fine-tuning. The use of PSO also enhances the robustness and generalization ability of the CNN model and its iterative nature allows for efficient discovery of hyperparameter similarity. The model was trained and tested on 13 different benchmark datasets and evaluated through six metrics: mean absolute error (MAE), mean square error (MSE), mean magnitude relative error (MMRE), root mean square error (RMSE), median magnitude relative error (MdMRE), and prediction accuracy (PRED). Comparative results reveal that the performance of the proposed model is better than other methods for all datasets and evaluation criteria. The results were very promising for predicting software cost estimation.

## Introduction

Today, the software industry and its associated technologies have become the language of the era, as they are involved in all industries and businesses. Therefore, software houses spread and the software industry became an essential matter. For this, the software cost estimation process is critical in software development and requires great experience from the company^1^.

Software cost estimation (SCE) involves estimating the effort, time, and resources needed to build an integrated software project by identifying customer requirements. This process is not limited to understanding user requirements specifications but also includes technical, software, and hardware requirements specifications in the initial stages^2^.

The software cost estimation process is considered an essential process for all companies due to its accuracy in cost estimation, which contributes to good planning and effective and distinguished project management by setting goals and deadlines for delivery, as well as the resources required to implement them. They also develop multiple alternatives that enable them to negotiate well and communicate effectively with clients by presenting multiple proposals, which contributes to gaining customers’ trust and satisfaction and ultimately leads to highly efficient and accurate programs^3^. It helps save resources, time, and budget needed for the project. It also allows project managers to allocate resources effectively, set realistic deadlines, and make sound decisions about project scope and objectives. It also contributes to budgeting and financial oversight, allowing organizations to create budgets and allocate funds appropriately, and it also shares insight into the expected costs of creating these projects. Its role is not limited to that, but rather it contributes to risk management by identifying and evaluating potential risks by considering several factors such as complexity, the technology used, and the experience of the work team. It serves as a light on the way to help decision-makers make informed choices. It helps in evaluating the feasibility and profitability of the project and return on investment^4^.

**Figure 1:**
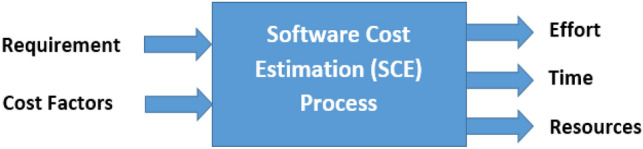
Software cost estimation process^5^.

The software cost estimation prediction process is based on historical data drawn from previous projects similar in operation to the projects want to build so that software houses can calculate the necessary cost. Therefore, the project requirements as well as its cost factors are required to be an input to that process to produce three outputs (effort, time, and resources) to implement the project, as shown in Fig. 1^5^.

Therefore, over the years, researchers have built many techniques, some of which are based on mathematical equations and called algorithmic methods, such as the construction cost model (COCOMO)^6^ and the software life cycle model (SLIM)^7^. Others rely on expert opinions called non-algorithmic methods, such as expert judgment^8^. Some relied on artificial intelligence techniques such as machine and deep learning algorithms. Some of them used machine learning techniques such as support vector regression (SVR)^9^, fuzzy logic^10^, and decision trees^11^. Others also relied on optimization algorithms such as the genetic algorithm^12^, the dolphin algorithm^13^, and the bat algorithm^14^. Many studies combined algorithms with a support vector regression (SVR) model based on fuzzy logic^15^. Deep learning techniques have also been used, such as neural networks^16–18^.

Despite the large number of studies that rely on artificial intelligence techniques, there is still an urgent need to use techniques that help improve forecasting and estimate software costs due to low forecast accuracy and high error rates^18,19^. Therefore, this study seeks to combine machine learning and deep learning techniques through the use of convolutional neural networks (CNN)^20^ and particle swarm optimization (PSO)^21^ algorithms to build a model to improve software cost estimation prediction.

The study map for this research is clear, as software cost estimation was drawn from the building stages of software development, which is a vital topic in software engineering^22^, in addition to the selection of convolutional neural networks, which are considered one of the most powerful branches of deep learning^23^. The particle swarm algorithm, which is an optimization algorithm and one of the most popular machine-learning techniques^24^, was also chosen. Deep and machine learning are artificial intelligence technologies that are considered powerful due to their enormous impact in various fields.

This study aims to use 13 datasets: COCOMO81, COCOMONasaV1, COCOMONasaV2, Desharnais, China, Albercht, Usp05, miyazaki94, Maxwell, Atkinson, Telecom, Kemerer, and Kitchengam were collected from the Promise repository^25^ and GitHub^26^ to build an intelligent model for software cost estimation prediction. Datasets were analyzed, processed, and visualized to understand and define them clearly. Time series forecasting^27^ was used to concatenate and process data into sequences to enable the application of a convolutional neural network. CNNs have shown notable success in various fields. By leveraging hierarchical layers of learnable filters, CNNs can extract high-level features from raw data, making them well-suited for analyzing complex datasets in software engineering. In addition to deep learning, optimization algorithms^28^ play a crucial role in tuning and improving the performance of CNN models. One such optimization algorithm is particle swarm optimization (PSO), a metaheuristic algorithm inspired by social behavior in nature. PSO iteratively explores the search space to find optimal solutions by simulating the behavior of birds or fish.

CNNs transform sequential data into a 2D or 3D network format, enabling the application of convolutional filters to extract features that define outputs or parameters. The study also distinguished itself in the use of the particle swarm optimization (PSO) algorithm as a next step, as it was combined with CNN. This allows the automatic adjustment of superior parameters, which reduces the manual effort of adjusting parameters. With its iterative nature, it allows the exploration of a variety of hyperparameter configurations which helps in efficient exploration of the parameter hyperspace. This contributes to improving the performance of the proposed model and raising its efficiency, and it also contributes to identifying the most relevant features. This in turn contributes to achieving the highest accuracy and optimal forecasting of cost estimation.

To ensure the effectiveness of the proposed model, six evaluation criteria are used: mean absolute error (MAE)^29^, mean squared error (MSE)^30^, mean magnitude relative error (MMRE)^31^, root mean square error (RMSE)^32^, median magnitude of relative error (MdMRE)^33^, and prediction accuracy (PRED)^34^.

The contributions of this study are distinct from previous studies as they contribute to:

- Propose a novel approach that combines CNN architecture and PSO optimization to predict software cost and effort estimation. This integration leverages the strengths of both deep learning and optimization algorithms to enhance the accuracy and reliability of cost estimation models.
- Develop a CNN base by designing a custom CNN architecture for cost estimation tasks. By adapting the architecture to the characteristics of software cost data, we aim to improve the model’s performance and generalization capabilities.
- Determine the ideal convolutional parameters, which in turn improves the performance and efficiency of the proposed model, by using PSO to determine the ideal hyperparameters for the CNN structure, including the learning rate, epochs, optimizer type, and batch size. This fine-tuning process improves model performance and convergence efficiency.
- Apply to several well-known datasets of different sizes, in addition to using six criteria to evaluate and prove the efficiency and effectiveness of the proposed model.
- The results of the study are very promising, as the results showed clear superiority compared to previous studies.

These contributions and promising results help determine the time, effort, and resources needed to develop a software project. Moreover, it instills confidence in software houses and stakeholders, enabling them to start their work on a solid foundation.

The rest of this article is organized as follows: Sect “Literature review” presents the literature review. Sect “The proposed methodology” describes the proposed methodology. Sect “Evaluation and comparison” elaborates on the evaluation and comparison. Sect “Conclusion” summarizes our findings and suggests future research directions.

## Literature review

During the past five decades, with the proliferation of the software industry, many researchers have sought to devise techniques that contribute to predicting software cost estimates. Researchers have devised three methods for predicting software cost estimates. Algorithmic methods are based on mathematical equations such as the construction cost model (COCOMO)^6^ and the software life cycle model (SLIM)^7^. Non-algorithmic methods rely on expert opinions such as expert judgment^8^. With the spread of technology over the past two decades and its reliance on artificial intelligence techniques, many researchers are constantly and permanently seeking to improve the accuracy of forecasting software cost estimates through the use of machine learning and deep learning techniques in predicting cost estimates. Many studies also contributed to this, such as support vector regression (SVR)^9^, fuzzy logic^10^, decision trees^11^, genetic algorithm^12^, dolphin algorithm^13^, and bat algorithm^14^. Studies also combined algorithms such as support vector regression (SVR) based on fuzzy logic^15^. Deep learning techniques have also been used, such as neural networks^16–18^.

Sivanageswara et al^35^ presented a model based on the multi-objective practical swarm algorithm for parameter tuning. The COCOMO dataset was used to train and test the proposed model. The model was evaluated using the mean absolute relative error (MARE) and prediction evaluation criteria. The results were somewhat acceptable, as the MARE and prediction values for the proposed model were 9.0143 and 24% respectively.

Idri et al.^36^ presented a model to predict the effort, cost, and resources required for any production project framework. The model was designed by combining the principles of the ID3 decision tree with fuzzy theoretical concepts, which enables the model to deal with imprecise data. The model was evaluated using MMRE and PRED prediction criteria on the Tukutuku dataset. The results were compared with three other versions of decision trees, namely ID3, C4.5, and CART. The proposed model has proven promising results compared to others in its role of contributing to preparing budgets, examining risks, arranging and monitoring projects, and investigating changing task perceptions.

Reddy et al.^37^ proposed a multi-layer neural network model by iteratively processing a set of training samples and COCOMO datasets were used to train and test the network. The model was evaluated using the mean magnitude of relative error and compared with the results of the COCOMO model. The proposed model showed better results compared to other models, where the estimated effort is closer to the actual effort.

Fadhil et al.^13^ presented a model based on the dolphin swarm algorithm and the hybrid bat (DolBat) algorithm to improve cost estimation models. The dolphin swarm algorithm is particularly suitable for optimization tasks, with fewer individuals, more fitness function calls, and taking advantage of echolocation to obtain the solution more effectively. This study was conducted on COCOMOnasaV1 and COCOMOnasaV2 datasets. The model was evaluated using the MMRE metric and was compared with other algorithms such as algorithmic genetics. The proposed model showed superiority over other algorithms.

Vo van et al.^38^ proposed a model to evaluate the impact of data aggregation on estimating software development efforts and then find the best aggregation method. The model was called the effort estimation using the machine learning applied to clusters model (EEAC). This study evaluated by mean absolute percentage error (MAPE), root mean square error (RMSE), mean absolute error (MAE), mean balance relative error (MBRE), and mean inverted balance relative error (MIBRE). Experimental results show that the estimation accuracy obtained through clustering consistently outperforms the accuracy without clustering for both function point analysis (FPA) and EEAC methods. The average improvement rate from using grouping rather than non-grouping was highest at 58.06%, according to RMSE. By the EEAC method, this number reached 65.53%. The categorical variable for the industry sector yields the best estimate of precision compared to the other clustering criteria and k-means clustering. The improvement in accuracy in terms of RMSE when applying this standard is 63.68% for the FPA method and 72.02% for the EEAC method.

Rahman et al.^39^ presented a comparison between three software cost estimation forecasting algorithms, namely decision tree, support vector regression (SVR), and K-nearest neighbor regression (KNN), where he processed and analyzed the datasets and applied the proposed algorithms to them. To evaluate the proposed model, he used three evaluation criteria: mean absolute error (MAE), mean square error (MSE), and R square. The study showed the superiority of decision trees over other algorithms.

Alhazmi et al.^40^ used bagged learning with base learner linear regression, SMOReg, MLP, random forest, REPTree, and M5Rule to predict software cost estimation and implemented a feature selection algorithm to examine the effect of the BestFit feature selection algorithm and genetic algorithm. They used a China dataset to evaluate this. The results show that the average relative error size of the M5 packing rule with the genetic algorithm as feature selection is 10%, which makes it better than other algorithms.

Ali et al.^41^ presented an estimation model for a heterogeneous group effort. The proposed model consists of independent estimation models such as use case point, expert judgment, artificial neural network, and linear clustering rules, and the effort of each basic model is combined. The model was applied to the ISBSG dataset using three shapes to avoid bias. The results showed that the ensemble technique gave better estimation results compared to the received estimation strategies.

Varshini et al.^42^ presented single and combined techniques, which are the combination of single techniques. They used random forest, support vector machine, decision tree, stacking using support vector machine, and stacking using random forest. The experiment was conducted on datasets and the model was compared using the datasets of Brecht, China, Descharnais, Kemmerer, Kitchenham, Maxwell, and Kokomo81. The model was evaluated using mean absolute error (MAE), root mean square error (RMSE), and R-squared. The results showed the superiority of the random forest compared to other models, such as machine learning algorithms and other clustering techniques.

Zakaria et al.^43^ presented a model based on support vector machine and linear regression machine learning algorithms to estimate software cost. An application known as SOFREST Estimator has been developed. Four algorithms, namely random forest, regression tree, linear regression, and support vector machine, were applied to the COCOMONasaV1, COCOMONasaV2, and COCOMO81 datasets. The model was evaluated using several evaluation criteria, namely mean squared error, root means squared error, mean absolute error, mean magnitude of relative error, min–max accuracy, correlation accuracy, and P-value, and the results showed the superiority of the targeted algorithms with the datasets. The experiment showed good performance and high accuracy of the vector machine support algorithm in estimating programming effort.

Sharma et al.^17^ provided four different methods for the software cost estimation prediction namely: localized neighborhood mutual information based neural network (LNI-NN)^44^, fuzzy-based neural network (NFL)^45^, adaptive GA-based neural network (AGANN)^46^, and GEHO-based NFN (GEHO-NN)^47^. The models were applied to five datasets: COCOMO81, COCOMONasaV1, COCOMONasaV2, China, and Desharnais, and evaluated through four prediction metrics: mean magnitude relative error (MMRE)^31^, root mean square error (RMSE)^32^, median magnitude of relative error (MdMRE)^33^, and prediction accuracy (PRED)^34^. The findings indicated a modest improvement in the prediction rates of software estimation.

Kassaymeh et al.^18^ presented a model to evaluate a software cost estimation model using a fully connected neural network (FCNN) model^48^ and a gray wolf optimizer (GWO)^49^, called GWO-FC, and tested the model on 12 datasets. Using several evaluation criteria, namely: mean square error (MSE), relative absolute error (RAE), mean absolute error (MAE), variance accounted for (VAF), Manhattan distance (MD), root mean square error (RMSE), and relative square error. root mean (RRSE), mean relative error (MdMRE), correlation coefficient (R2), Euclidean distance (ED), standard accuracy (SA), and effect size (4). The results showed the clear superiority of the proposed model for some datasets, but some datasets had similar results.

Table 1 has been added to summarize what has been achieved in previous studies on software cost estimation. Based on what was found in previous studies, the review showed that the prediction accuracy of software cost estimation is neither stable nor effective, as the prediction error rates were very high and the prediction accuracy was low^18,19^. Also, most studies used a small number of datasets and evaluation criteria to train and test models. Therefore, this study aims to build a model that can predict cost estimation effectively and with high efficiency. Therefore, the proposed study focuses on software cost estimation prediction on 13 datasets. It is also distinguished from previous studies by its superior ability to process data and present it smoothly, as well as using time series prediction and finding correlations between features to apply a convolutional neural network aimed at extracting features. The study was not limited to that, but rather combined CNN and the particle swarm algorithm to work on improving the values of superior parameters and allowing them to be automatically tuned and modified. It also allows improved predictive performance on non-automatic data, and the iterative nature of the optimization algorithm allows exploration of the hyperparameter space. The study is distinguished from its predecessor not only in covering a large number of datasets for forecasting but also in using six criteria to evaluate the model, which contributes to improving the efficiency and effectiveness of prediction.

**Table 1:** Comparison between the literature review studies.

Comparison	Algorithm	Datasets	Evaluation criteria	Strengths	Limitations
Sivanageswara et al.^35^	Multi-objective practical swarm algorithm	COCOMO	MARE, prediction	The study sought to apply the multi-objective technique, and it is one of the first studies that focused on the use of multi-objective technique to reduce the error size and increase prediction rates.	Despite its clear goal, the prediction rates were not high and its results did not achieve the desired goal. It has not been tested except on one dataset. The model was evaluated with only two evaluation criteria, which is very few.
Idri et al.^36^	Fuzzy decision tree	Tukutuku	MMRE, prediction	The study showed excellent results compared to others, as the prediction percentage was 98.11, which was unprecedented.	It is used only for web applications. The model was applied to only one dataset, which is not enough to predict the accuracy of the model. Only two evaluation criteria were used.
Reddy et al.^37^	Multi-layer feed-forward neural network	COCOMO	MMRE	It uses a minimal number of layers and nodes.	The study used only one dataset for this. Only one evaluation criterion and this will not sufficiently support accuracy. The results were acceptable but not outstanding.
Fadhil et al.^13^	Dolphin swarm algorithm and the hybrid bat (DolBat) algorithm	COCOMONasaV1, COCOMONasaV2	MMRE	The study is good in terms of combining the algorithms, and it also deliberately made a comparison between many other algorithms.	Two sets of data were not sufficient to prove the accuracy of the algorithm. Using only one evaluation criterion was not sufficient.
Vo van et al.^38^	Clustering the dataset through the k-means algorithm and applying the FPA and EEAC methods	ISBSG	RMSE, MAPE, MAE, MBRE, MIBRE	The study used several evaluation criteria to ensure the efficiency of the model and the step of using clustering algorithms in the field of software estimation.	Use only one dataset and it is not enough. There are other datasets ready to use and conduct experiments without the need for clustering because the dataset was already classified.
Rahman et al.^39^	Decision tree, support vector, regression, K-nearest neighbor regression	Created datasets by a software company called Edusoft consulted LTD	MAE, MSE, R Square	The distinguished study explained decision trees compared to other algorithms.	No comparison was made with previous studies in the same field. The datasets are new and we cannot access them. Use one new dataset even though there are others ready to use.
Alhazmi et al. ^40^	Genetic Algorithm, Linear regression, SMOReg, MLP, random forest, REPTree, M5Rule	China	MMRE	The study used several algorithms to predict cost estimates, and the genetic algorithm proved superior to other algorithms.	Only one dataset and one evaluation criteria were used, which are not sufficient to prove the efficiency of any of the algorithms used in the prediction.
Ali et al.^41^	Case point, expert judgment, artificial neural network	ISBSG	MMRE, PRED	Linking heterogeneous technologies has the role of establishing good alternatives for future evaluation. The use of multiple estimation methods in one study is important and new. Using multiple estimation methods in one study is important and new.	They were tested on only one dataset to evaluate the model and were not compared to other works to show their superiority or strength.
Varshini et al.^42^	Random forest, support vector machine, decision tree, stacking using support vector machine, stacking using random forest	Brecht, China, Descharnais, Kemmerer, Kitchenham, Maxwell, Kokomo81	MAE, RMSE, R square	A large number of algorithms were used to compare them, in addition to numerous datasets during the study.	Only three criteria were used to evaluate the study, which is not sufficient. In addition, the results were not stable and satisfactory enough.
Zakaria et al.^43^	Random forest, regression tree, linear regression, support vector machine	COCOMONasaV1, COCOMONasaV2, COCOMO81	MSE, RMSE, MAE, MMRE, P-value, min–max accuracy	The study used many evaluation criteria to prove the efficiency of the model.	The study used only three datasets, and this is a small number to prove the efficiency of the study.
Sharma et al.^17^	LNI-NN, fuzzy-based neural network, adaptive GA-based neural network, GEHO-based NFN	COCOMO81, COCOMONasaV1, COCOMONasaV2, China, Desharnais	MMRE, RMSE, MdMRE, PRED	The study used five datasets of different sizes to prove the efficiency of the algorithm and use modern algorithms to provide better results.	Only four criteria were used for evaluation. It was possible to use a larger number to prove higher efficiency. Despite the use of modern algorithms, the prediction accuracy is low and unstable.
Kassaymeh et al.^18^	A fully connected neural network (FCNN) and a gray wolf optimizer (GWO), called GWO-FC	Albrecht, COCOMO81, COCOMONasaV1, COCOMONasaV2, China, Desharnais, Cosmic, Kemerer, Kitchenham, Maxwell, Miyazaki 94, USP05	MSE, RAE, MAE, VAF, MD, RMSE, RRSE, MdMRE, R2, ED, SA, Effect size	The study combined machine learning networks and deep learning through neural networks and gray wolf optimizer. The experiment was also conducted on 12 datasets in addition to 12 evaluation criteria.	The experience was wonderful and the results were distinctive for most datasets, but they were normal or acceptable for others, that is, they were not at the same level of excellence for all datasets. The PRED measure was not used, which is one of the most famous software engineering measures and perhaps highlights the results more clearly.

## The proposed methodology

**Figure 2:**
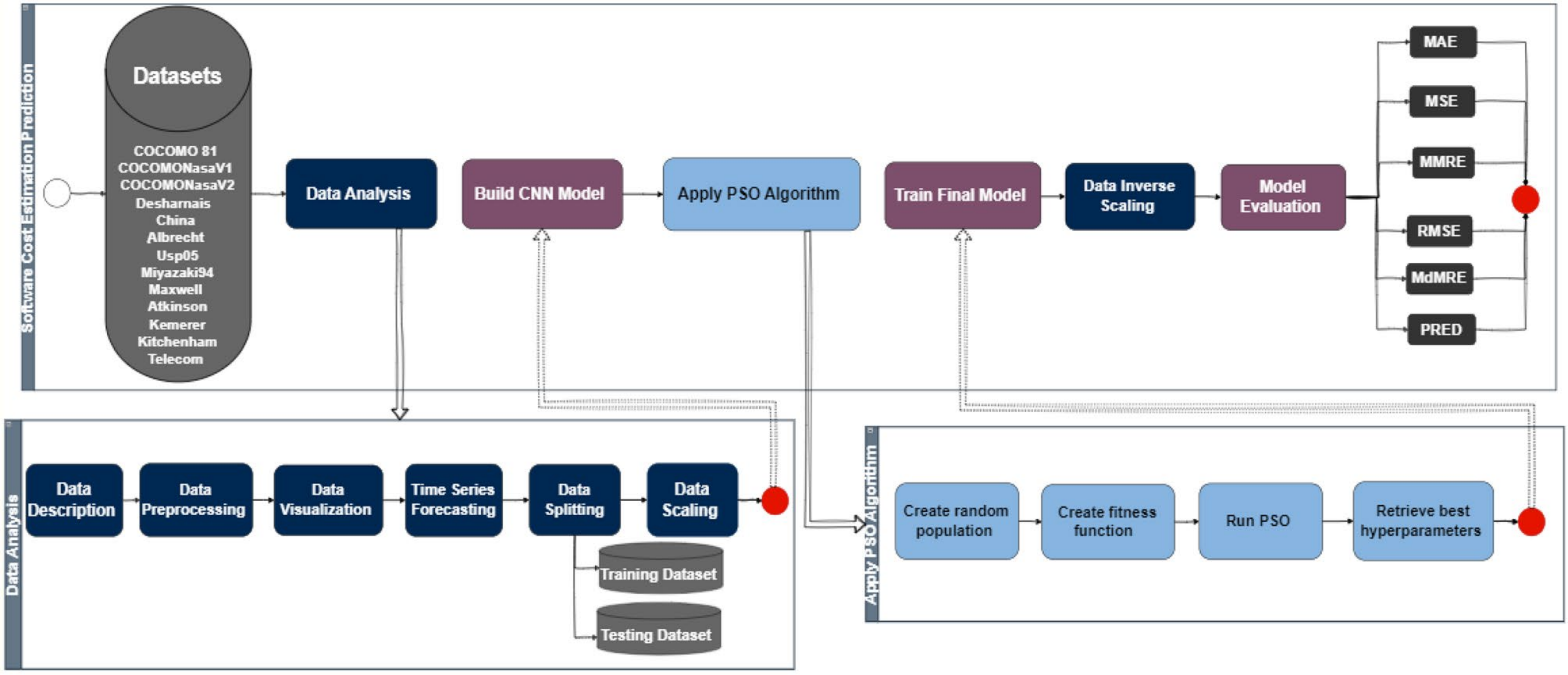
The proposed SCE model.

Researchers presented many studies that contribute to predicting software cost estimates, but their results were not accurate or satisfactory enough, so the study sought to build a model based on artificial intelligence techniques in its mechanisms. The study sought to use machine learning and deep learning techniques by using a convolutional neural network to predict software cost estimates and improve network performance through particle swarms for optimization, which in turn contributes to parameter optimization. The proposed study includes an organized and interconnected series of procedural steps that aim to enhance reliable predictive results, as shown in Fig. 2.

The process entails collecting permanent datasets through a systematic data collection phase. Next, it involves comprehensive data analysis, including descriptive statistics, preprocessing techniques, exploratory data visualization, and identification of distinct training and test sets, culminating in data normalization through scaling procedures. The subsequent phase involves the custom development of a CNN architecture specifically designed for cost estimation purposes. After the architecture is designed, the PSO is set to optimize the hyperparameters, including iterative parameter generation, fitness function formulation, algorithmic implementation, and optimal hyperparameter extraction, thus enhancing the CNN performance. After hyperparameter optimization, rigorous training of the model is performed using the custom training dataset. When training is finished, the normalization process is reversed to restore the data to its original state. The final evaluation of model performance comes from a comprehensive evaluation that uses six distinct evaluation metrics, including mean absolute error (MAE)^29^, mean square error (MSE)^30^, root mean square error (RMSE)^31^, mean magnitude of relative error (MMRE)^32^, median magnitude relative error (MDMRE)^33^, and prediction accuracy (PRED)^34^. This rigorous methodological framework represents a scientific endeavor aimed at enhancing the accuracy and reliability of software cost estimation models.

Implementing the proposed methodology for software cost estimation is intertwined with several challenges, each of which necessitates comprehensive examination to ensure its effectiveness and robustness in practical contexts. These challenges span diverse dimensions, each of which requires careful attention and strategic mitigation strategies to support the credibility and applicability of the methodology. The challenge of data availability and quality is that procuring comprehensive and unbiased datasets is extremely challenging, given the inherent variability and potential biases in data sources. Data quality issues, such as incompleteness or skewed distributions, greatly impact the accuracy and generalizability of predictive models and the challenge has been addressed by using data scaling to keep data within a single context and have close, eligible values during processing.

The second challenge is computational complexity, as combining a convolutional neural network (CNN) architecture with particle swarm optimization (PSO) introduces significant computational requirements, especially during hyperparameter optimization. It requires effective management of computational complexity. The challenge was met by customizing optimization strategies to ensure practical feasibility and scalability. The problem of sensitivity of hyperparameter tuning also arises, in which hyperparameter tuning using PSO is vulnerable to changes in initial conditions, which emphasizes the importance of rigorous experimentation and validation procedures. The use of powerful optimization algorithms and validation techniques is essential to achieve stable and optimal model performance amid hyperparameter sensitivity. It also shows the challenge of overfitting and generalization in which achieving a careful balance between model complexity and generalization performance is crucial to mitigate overfitting. Techniques such as regularization and cross-validation serve as indispensable tools to ensure that developed models generalize well to unseen data while overfitting challenges. Comprehensively addressing these challenges is crucial to enhancing the credibility and practical utility of the proposed methodology in software cost estimation research endeavors.

### Datasets

It was based on 13 benchmark datasets that are publicly available and easily accessible via the Promise^25^ and GitHub^26^ repositories. These datasets are specifically used to estimate the effort and cost of software and have a close relationship with the literature, as all previous studies depend on them, so they are suitable for evaluating the proposed model. Their sizes range from small to medium to large to suit all types of projects that the model can predict. Its features range from 6 as the minimum number of features used to 24 features as the largest number of features to train the proposed model. This adds great diversity to prove the efficiency of the proposed model, as shown in Table 2 which is not limited to that only, but provides several columns representing the name of the datasets used, the source of the repository from which the datasets were collected, the number of projects for each project, in addition to the number of features, the number of missing values, and the effort output of each dataset, whether monthly or hourly, through which the cost is estimated. Software. It is clear from seeing the statistics that there is a difference in the size of the data, as Albrecht, Kemmerer, and Miyazaki are considered small datasets, while China, Kokomo, Maxwell, and Kokomo are medium and large datasets.

**Table 2:** Statistics of the datasets.

Datasets	Source repository	No. of projects	No. of features	No. of missing values	Output attribute-effort (Unit)
COCOMO81	PROMISE	63	17	0	Person-month
COCOMONasaV1	PROMISE	60	17	0	Person-month
COCOMONasaV2	PROMISE	93	24	0	Person-month
China	PROMISE	499	15	0	Person- hours
Desharnais	PROMISE	81	10	4	Person- hours
Albrecht	PROMISE	24	8	0	Person-month
Usp05	GitHub	203	8	2	Person-month
Miyazaki94	GitHub	48	8	0	Person-month
Maxwell	GitHub	62	23	0	Person- hours
Atkinson	GitHub	16	15	0	Person-month
Kemerer	GitHub	15	6	0	Person-month
Kitchenham	GitHub	145	7	2	Person-month
Telecom	GitHub	18	3	0	Person-month

### Data analysis

#### Data description

Firstly, datasets are uploaded to Google Drive as data files and then read and added through Google Colab^50^. This study uses the read CSV() function to read those files. The info() function is also used to find out all the information about the data used, such as the number of rows and columns and whether there are null values. To understand the data statistically and distribute it, this study uses the describe() function to standardize the values and ensure that they are all numerical. At the end of the analysis process, a copy of the datasets is made to be used in that process to preserve the basic data. To complete the process, use the copy () function.

#### Data preprocessing

After reading the data, the preprocessing phase begins to eliminate errors, inconsistencies, missing values, as well as outliers^51^. First, all values are converted to numerical, as the dev_mode attribute is converted from object to numerical. The data is also converted sequentially to be linked, with the time attribute set as an index for the data sequence. In addition, the absence of null values is also checked by the isnull().sum() function.

#### Data visualization

Data is represented to determine the extent of communication and interrelation between that data. Each attribute is represented by two horizontal and vertical axes, representing the extent of correlation between these variables^52^. This is reported by the corr() function. To produce a set of values in a certain range. If the result between the correlations of the two variables is equal to one, this means that the relationship between them is very strong. If it is equal to zero, it means that there is no relationship between them. However, if the result is a negative value, the relationship between them is inverse.

#### Time series forecasting

Time series forecasting is a method of predicting future values based on previous observations of the variable and includes analysis of historical data to identify patterns and trends that can then be used to build a forecasting model. It includes several common methods, including neural networks used in the proposed model. It is widely used in finance, economics, weather forecasting, and many other fields. It is very important to evaluate the performance of the chosen method and take into account factors such as data quality and seasonality when performing time series forecasting^53^.

Time series forecasting begins with the preprocessing phase of critical data, which identifies the time dimension as a pivotal factor in cost analysis. By converting the time column to date-time format and then indexing the data accordingly, the chronological order of the program cost data is preserved, laying the foundation for rigorous time series analysis. Through techniques ranging from traditional statistical methods to advanced machine learning algorithms, historical cost trends are leveraged to forecast future spending patterns. This predictability empowers project stakeholders with proactive decision-making tools, enabling them to overcome the uncertainties inherent in software development projects. Time series forecasting is emerging as an indispensable tool in the software cost estimation arsenal, providing a data-driven approach to enhance project planning and execution.

#### Data splitting

This process is also called data partitioning. The data is divided into distinct groups for specific purposes. In this study, it is divided by 20–80% to be the largest percentage for the training process, where the model is trained on that data. The remaining 20% of the data is used in the testing process to measure the accuracy of the proposed model.

#### Data scaling

It is also called the process of data normalization or standardization which is used to convert the numeric features of a dataset to a common scale. This method is used so that the algorithm can handle all values and not ignore them, especially if they are high values.

### Build CNN model

**Figure 3:**
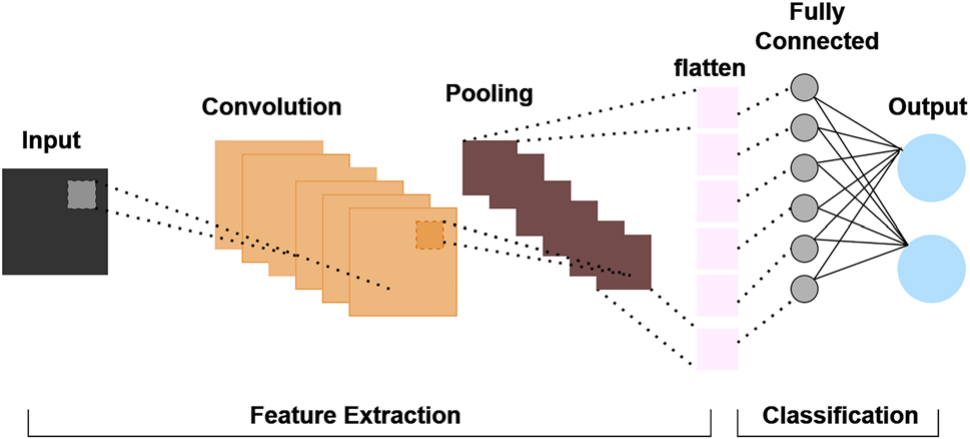
CNN architecture^55^.

Convolutional neural networks (CNNs) represent a massive class of deep learning models precisely designed to address the complex challenges inherent in analyzing images, video, and digital data such as time series or tables. These networks are characterized by a multi-layer architecture, comprising complex arrangements of convolutional layers, pooling layers, and densely connected layers, which are collectively designed to facilitate the extraction and interpretation of complex patterns. CNNs have emerged as a cornerstone, capturing the attention of researchers and achieving groundbreaking advances in data processing^20^. The central layers of the CNN framework, as shown in Fig. 3, lie in the convolutional layers, which serve as the underlying mechanism for extracting features from the raw input data. These layers work similarly to a complex set of digital filters, carefully examining the input and distinguishing the signals. Through a process of mathematical convolution, each convolutional layer produces a feature map, encapsulating salient aspects of the input content. Moreover, the subsequent integration of pooling layers within the CNN architecture facilitates hierarchical organization and densification of extracted features, thus enhancing computational efficiency and enabling robust representation learning. At their core, CNNs embody a cutting-edge blend of mathematical abstraction and computational prowess, revolutionizing the AI-powered data analysis landscape^54^.

Mathematically, the convolution operation within a CNN is denoted as in Eq. (1):

1 $$\left( {I*K} \right)\left( {p, q} \right) = \Sigma \Sigma I\left( {m, n} \right)K\left( {p - m, q - n} \right)$$

where I represents the input, K denotes the kernel, and * signifies the 2D convolution operation, with (p, q) denoting the position within the resulting feature map.

In the convolutional layer, the resultant feature map C is computed as in Eq. (2):

2 $$C\left( {p,q} \right) = f\left( {\Sigma \Sigma K\left( {m,n} \right)I\left( {p - m,q - n} \right) + b} \right)$$

where f represents the activation function, such as rectified linear unit (ReLU), and b signifies the bias term.

Pooling, a subsequent operation within CNNs, is described in Eq. (3):

3 $$P\left( {p, q} \right) = \max \left( {I\left( {m, n} \right)} \right) for m \in \left[ {r, r + s} \right), n \in \left[ {t, t + s} \right)$$

where P denotes the output of the pooling operation, while r and t represent the coordinates of the pooling window’s top-left corner, with s indicating the window size.

The fully connected layer, a pivotal component of CNNs, is articulated in Eq. (4):

4 $$Y = f\left( {WX + b} \right)$$

where Y signifies the output, W represents the weight matrix, X denotes the input vector, and b indicates the bias term.

**Figure 4:**
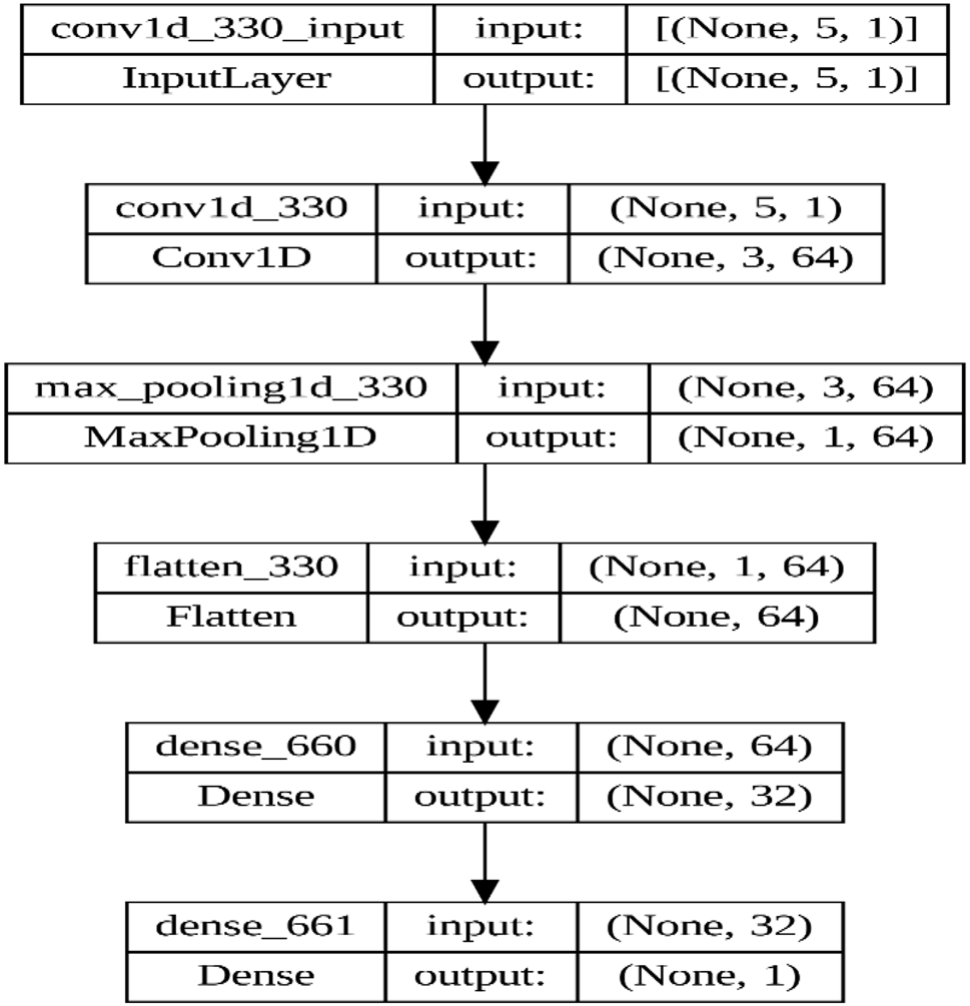
The visualization of the proposed CNN model.

When composing the neural network architecture, specific choices were made regarding the size of the network and the choice of activation functions based on experimental evidence and theoretical considerations. The proposed model is designed as a sequential set of layers within the tensorflow (tf) framework as in Fig. 4. It consists of the following layers^55–57^:

- Convolutional layer (Conv1D): this layer is generated with 64 filters and a kernel size of three, using a modified linear unit (ReLU) activation function. Previous research indicates that deeper networks with increased filtering capabilities can effectively capture complex patterns within time series data. Convolutional layers are adept at capturing local spatiotemporal patterns inherent in sequential data. The model can effectively extract diverse features while introducing the nonlinearity of the ReLU activation function needed to learn complex relationships within the data. The input format is specified as (n_steps, 1), where n_steps represents the number of time steps in the input data as it is represented by five steps. The input format is designed to suit the sequential nature of the data, ensuring compatibility with convolutional operation while encapsulating each data point as an individual feature.
- Pooling Layer (MaxPooling1D): a pooling layer with pool size two is applied after the convolutional layer to sample the feature maps. MaxPooling makes it easy to reduce spatial dimensions while preserving prominent features. Using pool size helps achieve computational efficiency and mitigates overfitting by retaining the most useful features.
- Normalization layer: this layer is responsible for normalizing multi-dimensional feature maps into a one-dimensional matrix, and preparing them for input into subsequent fully connected layers. Flattening allows subsequent fully connected layers to receive feature representations in a flattened format, which simplifies the process of extracting higher-level patterns from the data.
- Dense layers (fully connected layers): a dense layer containing 32 units and a ReLU activation function is used to transform the features. Fully connected layers capture general patterns within the learned features, enhancing the model’s ability to map features to the target variable. This increases the model's ability to distinguish complex patterns within the data.
- Output layer: the final output layer consists of a single module, suitable for regression tasks. A single output unit is well suited because it enables the model to directly predict continuous values, consistent with the goal of regression analysis.

This model architecture embodies a simplified approach to processing one-dimensional sequential data, such as time series, with a combination of convolutional and pooling layers for feature extraction and dimensionality reduction, followed by dense layers to represent higher-level features and regression outputs.

CNN is used in many fields as it is used in the software industry. Cong Pan et al.^58^ presented models based on it to predict software defects, find errors in programs, and prioritize testing efforts. Jian Li et al.^59^ also used it to predict software defects, but he improved the model by linking it to the DP model to produce a CNN DP model that shows an improvement in results of 12% compared to previous studies. The model was also used to predict software defects and errors by Wongpheng et al.^60^, and experimental results showed that CNN was promising for predicting defects with an average accuracy of 70.2%. It has also been used to solve problems in software engineering, such as detecting code smells, which cause an increase in technical cost and negatively affect the software. Lui et al.^61^ used CNN techniques to detect code smells to improve accuracy and detect different types of smells.

### The particle swarm optimization (PSO) algorithm

Particle swarm optimization (PSO) algorithm is a stochastic population-based optimization technique that mimics the social behavior of a flock of birds or schooling fish and is particularly inspired by aggregation or swarming behavior observed in nature^21^. PSO was introduced by Kennedy and Eberhardt in 1995^62^ and has gained significant attention due to its simplicity and effectiveness in solving various optimization problems. In PSO, a set of candidate solutions, known as particles, iteratively searches the solution space guided by its own experiences and those of its neighbors. Each particle adjusts its position and velocity according to its best-known location and the best-known locations within its neighborhood. The movement of particles toward optimal solutions is governed by two main components: the cognitive component (exploitation), which represents the particle’s tendency to move toward its personal best solution, and the social component (exploration), which represents the particle’s tendency to move toward optimal solutions. The best solution worldwide. Found by her neighbors. This balance between exploration and exploitation enables PSO to efficiently explore the solution space while converging toward promising regions^63^.

PSO dynamics are governed by various parameters such as swarm size, inertial weight, cognitive and social learning factors, and topology that determine the neighborhood structure. These parameters play critical roles in determining an algorithm’s convergence speed, its exploration–exploitation trade-off, and its robustness in different problem domains. Extensive research has been conducted to study the effect of parameter settings and propose adaptive strategies to adjust these parameters during the optimization process dynamically. In addition, several variants and extensions of PSO have been proposed to enhance its performance, including hybrid approaches that combine PSO with other metaheuristic techniques, constraint-handling mechanisms, and parallel implementations^64^.

In PSO, each potential solution, known as a particle, adjusts its position and velocity in the search space based on its own experience and the experiences of its neighbors. The position of particle *i* at iteration t, as in Fig. 4, is represented by ${x}_{i}^{t}$. And its velocity by ${v}_{i}^{t}$. Each particle maintains its best-known position ${p}_{i}^{t}$, and the global best-known position among all particles is denoted as ${p}^{global}$.

The velocity update equation for particle *i* at iteration *t* in PSO is given by Eq. (5):

5 $$v_{i}^{t + 1} = w \cdot v_{i}^{t} + c1 \cdot r1 \cdot \left( {p_{i}^{t} - x_{i}^{t} } \right) + c2 \cdot r2 \cdot \left( {p^{global} - x_{i}^{t} } \right)$$

where w is the inertia weight controlling the impact of the previous velocity, c1 and c2 are the cognitive and social learning factors, controlling the particle’s tendency to follow its personal best and the global best position, respectively. r1 and r2 are random values drawn from a uniform distribution in the range [0, 1].

The position update equation for particle *i* at iteration *t* is then given by Eq. (6):

6 $$x_{i}^{(t + 1)} = x_{i}^{t} + v_{i}^{(t + 1)}$$

To update the personal best position (${p}_{{best}_{i}}$) of particle *i* and the global best position (${\text{g}}_{best}$), the following Eqs. (7), (8) are employed:

7 $$p_{{best_{i} }}^{t + 1} = \left\{ {\begin{array}{*{20}c} {p_{{best_{i} }}^{t} } & {if\, f\left( { p_{{best_{i} }}^{t} } \right) \le f\left( {p^{t + 1} } \right)} \\ {x_{i}^{t + 1} } & {if\, f\left( { p_{{best_{i} }}^{t} } \right) > f\left( { x_{i}^{t + 1} } \right)} \\ \end{array} } \right\}$$

8 $$g_{b} est^{(t + 1)} = \max \left\{ {f\left( y \right) , f\left( { g_{best}^{t} } \right)} \right\}where\, y \in \left\{ {p_{{best_{0} }}^{t} , p_{{best_{1} }}^{t} , \ldots , p_{{best_{n} }}^{t} } \right\}$$

**Figure 5:**
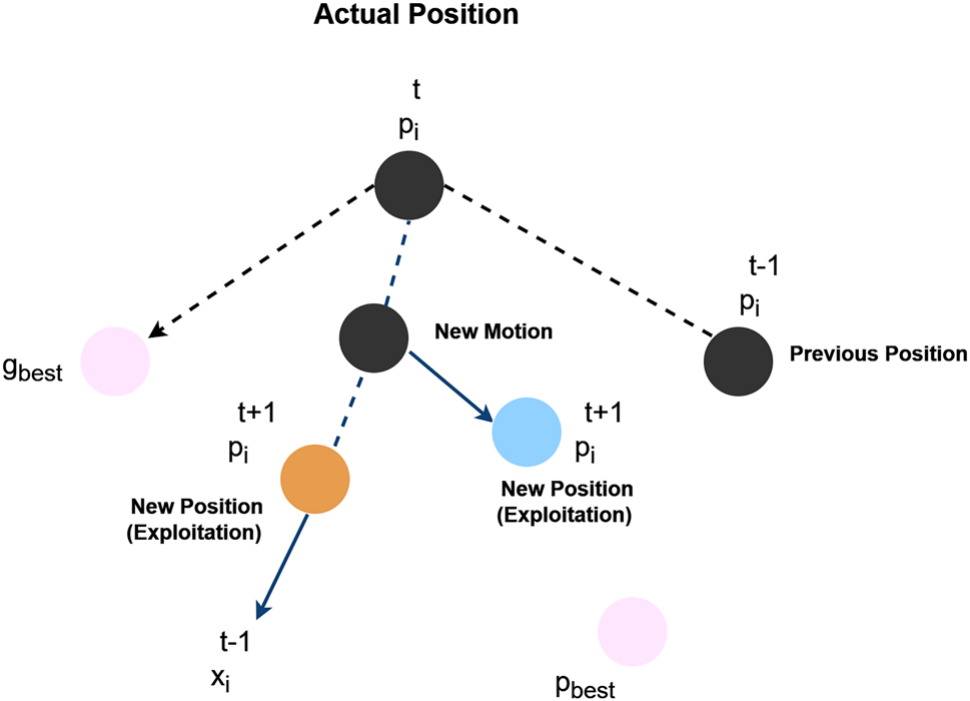
PSO movement.


Through these equations, particles iteratively explore the solution space, aiming to converge toward optimal solutions while balancing exploration and exploitation as shown in Fig. 5.

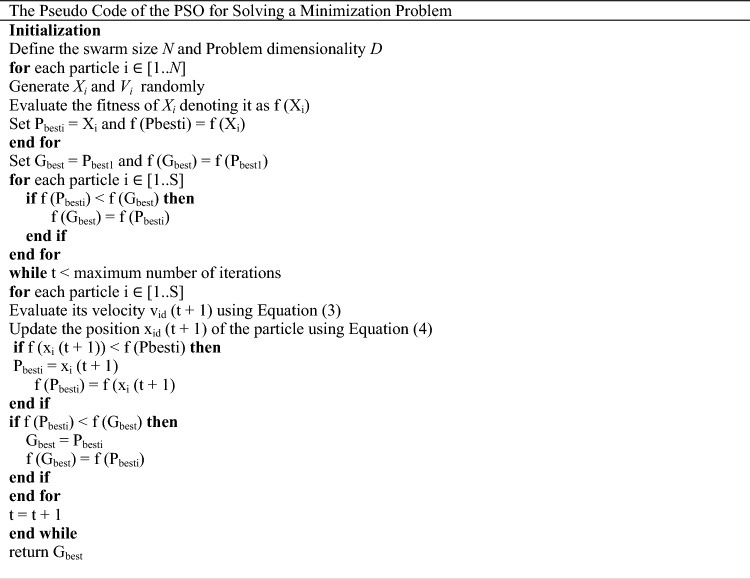


The presented pseudocode defines the operational framework of PSO to solve minimization problems. Its importance is multifaceted. Where you follow a set of steps^65^:

1. Structured initialization: the algorithm begins with a systematic initialization phase, where basic parameters such as swarm size and problem dimensions are determined. This phase sets the foundational framework for subsequent iterations.
2. Random generation of particle features: the pseudocode provides for the random generation of particle positions and velocities, a crucial step that imparts randomness to the algorithm, allowing exploration of the solution space.
3. Fitness evaluation: the fitness of each particle is evaluated based on its current position, providing a metric to measure its performance within the problem domain. This assessment is pivotal in directing the improvement process towards areas of improved fitness.
4. Dynamic updating of particles and global best positions: through iterative optimization, particles constantly update their personal best positions based on their fitness evaluations. At the same time, the algorithm dynamically adjusts the global best position, reflecting the collective optimization progress of all molecules.
5. Iterative optimization: the essence of the PSO algorithm lies in the iterative optimization process, where particles repeatedly adjust their velocities and positions to efficiently explore the solution space. This iterative optimization facilitates convergence toward optimal solutions over successive iterations.

PSO pseudocode serves as a foundational blueprint for developing effective optimization strategies, as its importance extends to its structured configuration, iterative optimization process, and applicability across diverse problem domains. Through its systematic approach to exploring and exploiting solution spaces, PSO contributes to the development of computational problem-solving methodologies.

The PSO algorithm has been used in similar studies, where Vahid et al.^66^ used it to estimate development efforts by combining it with Analogy-based estimation to increase the accuracy of estimating software development efforts. However, ABE is unable to produce accurate estimates when the level of importance of project features is not the same or when the relationship between features is difficult to determine. However, the results were promising by combining PSO and ABE and contributed significantly to the estimation.

Among similar studies, Windisch et al.^67^ presented a method for creating a test that achieves high coverage of structured code. PSO was used to search for relevant test cases. Experiments were conducted on 25 small industrial test objects and 13 more complex artificial test objects taken from different development projects. The algorithm has been shown to outperform most of the code elements to be covered in terms of effectiveness and efficiency.

#### Combining CNN with PSO

**Figure 6:**
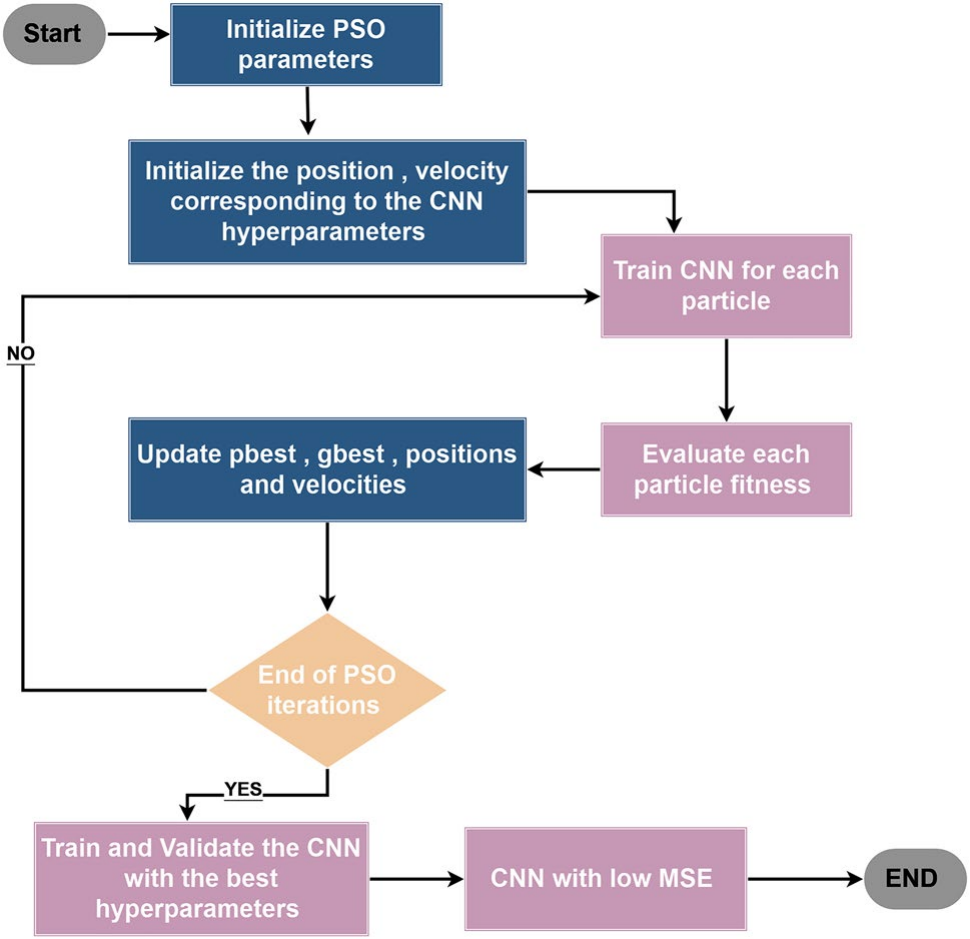
The flowchart of the combination of CNN with PSO.

The integration of particle swarm optimization (PSO) algorithm with convolutional neural networks (CNNs), as in Fig. 6, presents a distinctive approach for optimizing hyperparameters in machine learning tasks, particularly in the realm of time series forecasting. The fitness function, which encapsulates the performance evaluation of the CNN model, dynamically adjusts key parameters such as learning rate, number of epochs, optimizer type, and batch size, thereby facilitating the exploration of the hyperparameter space^68^.

In this combination, a CNN model is constructed using tensorflow’s Keras API, comprising layers tailored for time-series data analysis. Specifically, the model architecture includes a one-dimensional convolutional layer with 64 filters and ReLU activation, followed by max-pooling and flattening layers to extract relevant features. Subsequently, two dense layers with ReLU activation are added to facilitate learning, culminating in an output layer for regression tasks.

To optimize the model’s performance, PSO is employed to search for the optimal combination of hyperparameters. Through iterative updates guided by the fitness function, PSO navigates the hyperparameter space to identify configurations that minimize the validation loss, thereby enhancing the model's predictive accuracy and generalization capability^69^.

Upon completion of the PSO optimization process, the best-performing hyperparameters are retrieved for further analysis and model refinement. These hyperparameters, including the optimal learning rate, number of epochs, optimizer type, and batch size, are crucial determinants of the CNN model's efficacy in capturing intricate patterns within the time series data.

The fusion of PSO with CNNs offers several advantages in the context of time-series regression tasks. Firstly, it enables automated hyperparameter tuning, reducing the manual effort required for parameter selection and fine-tuning. Secondly, by exploring a diverse range of hyperparameter configurations, PSO enhances the robustness and generalization capability of the CNN model, leading to improved predictive performance on unseen data. Additionally, the iterative nature of PSO allows for efficient exploration of the hyperparameter space, facilitating the identification of optimal configurations within a reasonable computational budget.

The sequential implementation of the provided set is done through the definition of the fitness function, which serves as a corner reservation for evaluating the performance of the CNN. It accepts a set of parameters, including learning rate, epochs, optimizer type, and batch size, as input. The CNN model is created using tensorflow’s Keras API, which is precisely configured using layers specifically designed for analyzing time series data. The model undergoes clustering using the specified optimizer and loss function. Then, it undergoes a training phase using the provided training data (X_train and y_train) over a specified number of epochs and batch size. When the training phase is finished, the validation loss is calculated using the validation dataset (X_test and y_test), which serves as the fitness value to be minimized by the PSO algorithm.

The search bounds for hyperparameters are defined by defining lower and upper bounds for each hyperparameter (learning rate, epochs, optimizer type, batch size) to constrain the search space of PSO. PSO is called to search for the optimal set of hyperparameters that minimizes validation loss. The pso function is used with the fitness function, lower and upper bounds, swarm size, and maximum number of iterations specified as arguments. Upon completion of the PSO optimization process, the best-performing parameters are extracted for further analysis and model improvement. These hyperparameters, including the optimal learning rate, epochs, optimizer type, and batch size, are recovered from the results obtained from the PSO optimization. The final 1D CNN model is trained and generated using the best hyperparameters obtained from the PSO optimization process. The model architecture reflects a predefined CNN architecture, including convolutional, max pooling, flat, and dense layers. These layers are configured based on optimal hyperparameters to ensure the model is effective in capturing complex patterns within the time series data. The model undergoes rigorous training to leverage the provided training dataset (X_train and y_train) to recognize and accommodate latent patterns and relationships within the data. In addition, the performance of the model is further evaluated using a validation dataset to ensure its generalizability and robustness.

The rationale for combining CNNs and PSOs stems from their proven effectiveness in similar studies. CNNs have shown great performance in various tasks including image recognition, natural language processing, and time series analysis. Its ability to automatically extract hierarchical features from data, especially in sequential data such as time series, makes it well suited for regression tasks such as software cost estimation. In addition, PSO has been widely used in optimization problems, including hyperparameter tuning of machine learning models. Their ability to efficiently explore large search spaces and avoid local optimization is consistent with the challenges posed by hyperparameter optimization in complex models such as CNNs. By leveraging the strengths of both CNN and PSO, the proposed approach aims to leverage their complementary capabilities to enhance the model’s predictive performance and convergence speed. Thus, the decision to combine CNNs with PSO is rooted in their established success in similar contexts and their potential synergy in addressing the specific challenges of hyperparameter optimization in predictive modeling tasks.

The optimization of hyperparameters is a crucial aspect in refining the performance of CNN models for regression tasks. Lower (lb) and upper (ub) bounds are established for parameters such as learning rate, epochs, optimizer type, and batch size, delineating feasible ranges for each parameter and guiding the optimization process. In this context, the lower bounds (lb) define the minimum acceptable values for hyperparameters, including a learning rate of 0.001, minimum of one epoch, and zero for the optimizer type (indicating the first optimizer in the list, Which is Adam optimizer), and a batch size of one. Conversely, the upper bounds (ub) specify the maximum acceptable values, encompassing a learning rate of 1.0, a maximum of 100 epochs, optimizer type 2 (indicating the last optimizer in the list, which is RMSprop optimizer), and a batch size of 100. Subsequently, PSO is employed to navigate this constrained parameter space and identify the optimal combination of hyperparameters. Configured with a swarm size of 30 and a maximum of 10 iterations, the PSO algorithm orchestrates an efficient search for the optimal hyperparameters, inspired by social behavior in nature. Upon completion, the best-performing hyperparameters, along with their associated fitness values, are extracted, furnishing valuable insights into the configuration that yields superior performance for CNN models across diverse regression datasets.

In this study, the application of PSO for hyperparameter tuning in CNNs has yielded promising results across thirteen datasets: COCOMO81, COCOMONasaV1, COCOMONasaV2, Desharnais, China, Albrecht, USP05, miyazaki94, Maxwell, Kemerer, Kitchenham, Atkinson and Teleecom dataset as shown in Table 3. The best hyperparameters retrieved by PSO for each dataset exhibit variations tailored to the unique characteristics of the data. Specifically, the optimal learning rate, number of epochs, optimizer type, and batch size have been determined to maximize the performance of the CNN model for regression tasks on these datasets.

**Table 3:** The best hyperparameters retrieved from PSO for the CNN model.

Dataset	Best Epochs	Best learning rate	Best batch size	Best optimizer
COCOMO81	52	0.19695964331885313	2	SGD
COCOMONasaV1	20	0.3606617606119912	13	RMS_prop
COCOMONasaV2	56	0.514062246058241	1	Adam
China	43	0.3260765812920042	24	RMS_prop
Desharnais	18	0.3216369132085036	82	Adam
Albrecht	100	0.001	1	Adam
USP05	6	1.0	63	SGD
Miyazaki94	20	0.5357781672926979	47	Adam
Maxwell	15	0.5798714106779497	32	SGD
Kemerer	97	0.2296028798361126	1	Adam
Kitchenham	20	0.7399659599666866	100	RMS_prop
Atkinson	92	0.07090338359805011	1	SGD
Telecom	84	0.42885135322158524	1	SGD

For instance, in the case of Albrecht, Atkinson, and COCOMO81, a relatively low learning rate combined with a moderate number of epochs and a suitable optimizer type has proven effective in achieving optimal results. On the other hand, for COCOMONasaV1, COCOMONasaV2, China, USP05, miyazaki94, Maxwell, and Kitchenham datasets, a higher learning rate coupled with a larger number of epochs and a specific optimizer type has demonstrated superior performance. The Desharnais and Kemerer datasets, known for their unique characteristics, necessitated a distinct set of hyperparameters to achieve optimal results, emphasizing the importance of dataset-specific tuning.

Furthermore, the application of PSO has enabled the identification of hyperparameters that facilitate efficient convergence and generalization, contributing to enhanced model performance across all datasets. The iterative optimization process guided by the fitness function has effectively navigated the hyperparameter space, leading to the discovery of configurations that minimize validation loss and improve the overall predictive accuracy of the CNN model.

#### Data inverse transformation

To use the datasets in the data analysis and processing phase, data scaling was performed, converting the numerical characteristics of the datasets to a common scale so that the model can handle all values and not ignore them, especially if the values are high^70^. After applying and training the model, the data is reverse transformed or data normalized, where the process returns the measured or transformed data to its original shape or size. This is done through data preprocessing techniques such as scaling or logarithmic transformation. The data inverse transformation is necessary to obtain the original data values for interpretation, analysis, or further processing. It is implemented through the inverseTransform() function.

## Evaluation and comparison

The evaluation process is the crucial step in the process of building the model to prove its efficiency and effectiveness for use. The proposed model is evaluated after training on test datasets to measure its accuracy and know the extent of its efficiency. To truly ensure its effectiveness, this section was divided into several sub-sections related to the specifications of the device used, as well as the evaluation criteria used, in addition to comparison with previous recent works, all of which discuss and compare the results.

### Experiment settings

The parameter configuration was the same for all over the experiment. The datasets were divided 80–20% for the training and testing phase. The population size of PSO was determined to be 50 and the maximum iterations were determined to be 10. Extensive experiments were performed to find the hyperparameter values and the optimal solution was chosen. The experiment was conducted on a PC with Intel Core i7 CPU, 64GB RAM, and NVIDIA GTX 1050i GPU, using Google Colab through Python and Google Drive.

### Evaluation criteria

Evaluating the proposed model is the most important step to ensure its effectiveness and efficiency of use. The model is evaluated using six common predictive criteria: mean absolute error (MAE)^29^, mean square error (MSE)^30^, mean magnitude of relative error (MMRE)^31^, root mean square error (RMSE)^32^, median of magnitude of relative errors (MdMRE)^33^, and predication accuracy (PRED)^34^.

#### Mean absolute error (MAE)

MAE is a measure commonly used to measure the mean squared difference between expected values and actual values by taking the mean of the squared differences. It provides a measure of the overall accuracy of the regression model. A lower value indicates better performance of the model. Where zero represents a perfect match between actual and expected values. It is widely used in the fields of machine learning, statistics, and data analysis to evaluate and compare different regression models^29^. It is calculated according to Eq. (9):

9 $$MAE = \frac{1}{n}*\sum\limits_{i = 1}^{n} {\left( {y - y^{\wedge}} \right)}$$

where n is the total number of observations, y is the actual value of sample i, and y^ is the prediction made by the model for sample i.

#### Mean magnitude of relative error (MMRE)

MMRE is one of the most popular metrics for predicting accuracy in software engineering. The average absolute percentage error between the actual and predicted values is calculated. It is calculated according to Eqs. (10), (11).

10 $$MRE = \frac{{\left| {Actual\, effort - Estimated\, effort} \right|}}{Actual\, effort} \times 100$$

11 $$MMRE = \frac{1}{M}\mathop \sum \limits_{1}^{M} MRE$$

where m is the total data points and ∑ denotes the sum of values in the entire dataset ^30^.

#### Mean square error (MSE)

A commonly used metric in statistics and machine learning to measure the mean squared difference between predicted values and actual values. In other words, it is a way to measure how far predicted values are from actual values. It is used in statistics and machine learning^31^. It is calculated through the Eq. (12):

12 $$MAE = \frac{1}{n}*\mathop \sum \limits_{i = 1}^{n} \left( {y - y^{\wedge}} \right)^{2}$$

where n is the total number of samples, y is the actual values, and y^ is the prediction made by the model for sample i.

#### Root mean square error (RMSE)

It is one of the most widely used metrics in forecasting. It is used to calculate the average size of the difference between actual and expected values. To calculate its value, you need the actual values and their corresponding expected values as shown in Eq. (13).

13 $$RMSE = \sqrt {\frac{{\sum \left( {P_{i} - Oi} \right)^{2} }}{n}}$$

where n is the total number of data points, P is the predicted value, O is the actual value, ^2 denotes the square of the difference, and represents the sum of the square differences in the datasets^32^.

#### Median of magnitude of relative errors (MdMRE)

One of the statistical measures used to evaluate forecast accuracy is used to calculate the absolute average^33^. It is calculated from the actual values and their corresponding predicted values by determining the relative error of each prediction by finding the absolute difference between the actual and predicted values using the formula. |(P−A)|/A. Then, arrange the relative errors in ascending order and calculate the average of the sorted relative errors as shown in Eq. (14).

14 $$MdMRE = median \left( {\left| {\frac{{y_{i} - \widehat{{y_{i} }}}}{{y_{i} }}} \right|} \right)$$

where ${y}_{i}$ is the actual value of the dependent variable for data point i, and $\widehat{{y}_{i}}$ is the predicted value of the dependent variable for data point i.

#### Predication accuracy (PRED)

PRED is a standard for prediction accuracy. One of the most widespread metrics in estimating the cost of software. It increases with improvement in model accuracy. It is calculated by the percentage of models in which the expected values match the actual values and is calculated through Eq. (15).

15 $$PRED = \frac{1}{n}\mathop \sum \limits_{i = 1}^{n} \left| {\frac{Estimation\, Effort - Actual\, Effort}{{Actual\, Effort}}} \right| K\%$$

where k% is the percentage error between actual estimation and effort estimation^43^.

### Experimental results

The proposed model was used to predict the software cost estimate due to the effectiveness of the algorithm in prediction and its accuracy, and it was evaluated according to the standards described in the previous section. Table 4 displays the results of the proposed model on the datasets.

**Table 4:** The results of the proposed model in terms of MAE, MSE, MMRE, RMSE, MdMRE, PRED.

Datasets	MAE	MSE	MMRE	RMSE	MdMRE	PRED
COCOMO81	0.00799	7.055e-05	0.02181	0.00839	0.01090	99.16
COCOMONasaV1	0.00739	6.084e-05	0.00879	0.00780	0.00439	99.22
COCOMONasaV2	0.00609	4.0960e-05	0.00350	0.00640	0.00175	99.36
China	0.00630	4.4890e-05	0.00028	0.00670	0.000140	99.33
Desharnais	0.00419	2.1160e-05	6.928e-05	0.00460	3.464e-05	99.53
Albrecht	0.00010	1.4400e-08	0.00308	0.00012	0.000128	99.98
Usp05	6.99e-05	8.1000e-09	0.002543	9.00e-05	0.001271	99.99
miyazaki94	9.99e-05	1.2099e-08	4.51e-05	0.00010	2.259e-05	99.98
Maxwell	5.00e-05	3.6000e-09	2.891e-06	6.000e-05	1.4459e-06	99.99
Atkinson	9.99e-06	3.999e-10	1.999e-06	5.783e-05	2.891e-06	99.99
Kemerer	2.00e-05	8.9999e-10	2.0713e-5	2.999e-05	1.0356e-05	99.99
Kitchenham	3.00e-05	1.5999e-09	8.510e-05	3.999e-05	4.2553e-05	99.99
Telecom	4.99e-05	3.5999e-09	1.240e-05	5.999e-05	1.2407e-05	99.99

The results shown in Table 4 indicate that combining machine learning and deep learning techniques in general under appropriate conditions and using techniques that contribute to improving performance, such as time series prediction, can contribute to producing very high results. Table 4 shows that the thirteen datasets excel in reducing the error rate during the prediction process and increasing prediction accuracy. The Usp05, Miyazaki94, and Atkinson datasets demonstrated unprecedented excellence according to the six criteria. The percentages for the rest of the datasets were also very promising, especially for the MSE metric in reducing error and the PRED metric in measuring the accuracy and efficiency of the proposed model.

### Comparison and discussion

These methods are evaluated by comparing them with other previous studies, as Sharma et al.^17^ applied his study on four algorithms: localized neighborhood mutual information-based neural network (LNI-NN), fuzzy-based neural network (NFL), adaptive GA-based neural network (AGANN) , and GEHO-based NFN (GEHO-NN) and Kassaymeh et al.^18^ applied his study on two algorithms: fully connected neural network (FCNN) and gray wolf optimizer (GWO) with fully connected neural network called GWO-FC. These studies were chosen for comparison due to its novelty and because the experiment was conducted on most of the datasets and used most of prediction criteria for evaluation. Rather, the proposed study used two more datasets and other evaluation criteria to confirm the accuracy, effectiveness, and efficiency of the proposed model, as shown in Table 5.

**Table 5:** Comparison between the proposed model and others.

Method	Metrics	MAE	MSE	MMRE	RMSE	MdMRE	PRED
LNI-based NN ^17^	COCOMO81	–	–	0.224	0.261	0.256	28.51
	COCOMONasaV1	–	–	0.243	0.183	0.249	50
	COCOMONasaV2	–	–	0.225	0.383	0.249	50
	China	–	–	0.240	0.148	0.255	44
	Desharnais	–	–	0.32	0.312	0.336	22.22
Neuro-fuzzy logic ^17^	COCOMO81	–	–	0.213	0.178	0.256	29.92
	COCOMONasaV1	–	–	0.236	0.131	0.215	62
	COCOMONasaV2	–	–	0.196	0.290	0.215	62
	China	–	–	0.220	0.075	0.240	70
	Desharnais	–	–	0.296	0.173	0.223	32
Adaptive GA-based NN ^17^	COCOMO81	–	–	0.199	0.130	0.235	46.15
	COCOMONasaV1	–	–	0.231	0.065	0.172	73.87
	COCOMONasaV2	–	–	0.174	0.232	0.172	70
	China	–	–	0.192	0.056	0.218	76
	Desharnais	–	–	0.197	0.111	0.181	47.05
GEHO-based NFN ^17^	COCOMO81	–	–	0.174	0.055	0.223	57.14
	COCOMONasaV1	–	–	0.220	0.060	0.130	83.14
	COCOMONasaV2	–	–	0.128	0.960	0.130	83.14
	China	–	–	0.167	0.39	0.168	84
	Desharnais	–	–	0.112	0.060	0.100	88.23
FCNN ^18^	COCOMO81	0.1518	3.19E-02	–	0.1787	0.1725	–
	COCOMONasaV1	0.1063	2.53E-02	–	0.1591	0.7871	–
	COCOMONasaV2	0.0967	2.01E-02	–	0.1418	0.8245	–
	China	0.0310	2.88E-03	–	0.0536	0.5708	–
	Desharnais	0.1268	3.47E-02	–	0.1862	0.4391	–
	Albrecht	0.1180	3.12E-02	–	0.1767	0.0652	–
	Usp05	0.0762	1.21E-02	–	0.1098	3.3709	–
	Miyazaki94	0.0709	1.11E-02	–	0.1054	0.5017	–
	Maxwell	0.1157	1.77E-02	–	0.1333	0.6550	–
	Kemerer	0.2021	5.87E-02	–	0.2423	0.2926	–
	Kitchenham	0.0981	3.38E-02	–	0.1839	0.4104	–
GWO-FC ^18^	COCOMO81	0.0130	2.82E-04	–	0.0168	0.0131	–
	COCOMONasaV1	0.0048	4.58E-05	–	**0.0068**	0.0063	–
	COCOMONasaV2	0.0653	1.17E-02	–	0.1082	0.6308	–
	China	0.0218	1.34E-03	–	0.0366	0.3815	–
	Desharnais	0.0321	1.85E-03	–	0.0430	0.0843	–
	Albrecht	0.0006	4.57E-07	–	0.0007	0.0011	–
	Usp05	0.0422	4.64E-03	–	0.0681	2.7289	–
	Miyazaki94	0.0509	3.47E-03	–	0.0589	0.5583	–
	Maxwell	0.0037	3.15E-05	–	0.0056	0.0246	–
	Kemerer	0.0003	8.81E-08	–	0.0003	0.0008	–
	Kitchenham	0.0615	6.12E-03	–	0.0783	0.4622	–
The proposed model	COCOMO81	**0.00799**	**7.055e-05**	**0.02181**	**0.00839**	**0.01090**	**99.16**
	COCOMONasaV1	**0.00739**	**6.084e-05**	**0.00879**	0.00780	**0.00439**	**99.22**
	COCOMONasaV2	**0.00609**	**4.0960e-05**	**0.00350**	**0.00640**	**0.00175**	**99.36**
	China	**0.00630**	**4.4890e-05**	**0.00028**	**0.00670**	**0.000140**	**99.33**
	Desharnais	**0.00419**	**2.1160e-05**	**6.928e-05**	**0.004600**	**3.464e-05**	**99.53**
	Albrecht	**0.00010**	**1.4400e-08**	**0.00308**	**0.00012**	**0.000128**	**99.98**
	Usp05	**6.99e-05**	**8.1000e-09**	**0.002543**	**9.00e-05**	**0.001271**	**99.99**
	Miyazaki94	**9.99e-05**	**1.2099e-08**	**4.51e-05**	**0.00010**	**2.259e-05**	**99.98**
	Maxwell	**5.00e-05**	**3.6000e-09**	**2.891e-06**	**6.000e-05**	**1.4459e-06**	**99.99**
	Atkinson	**9.99e-06**	**3.999e-10**	**1.999e-06**	**5.783e-05**	**2.891e-06**	**99.99**
	Kemerer	**2.00e-05**	**8.9999e-10**	**2.071e-05**	**2.999e-05**	**1.0356e-05**	**99.99**
	Kitchenham	**3.00e-05**	**1.5999e-09**	**8.510e-05**	**3.999e-05**	**4.2553e-05**	**99.99**
	Telecom	**4.99e-05**	**3.5999e-09**	**1.240e-05**	**5.999e-05**	**1.2407e-05**	**99.99**

Significant values are given in bold.

Table 5 shows the comparison between the proposed model and the previous studies that were applied during 2022 and 2023, and the six criteria were applied. The highest results are marked with a bold to show the superiority of the proposed model. The results on the MMRE metric for the first four studies, LNI-based NN, Neuro-fuzzy logic, adaptive GA-based NN, and GEHO-based NFN which applied in Sharma et al.^17^, showed that the five datasets in the proposed model excelled in reducing the error rate, especially in the China and Desharnais datasets. While the datasets showed very good ratios using the RMSE measure. The results were also promising for the datasets on MdMRE metric for the China and Desharnais datasets were similar to the COCOMONasaV1 and COCOMONasaV2 datasets. While the results of the PRED metric for the five datasets were very distinctive and promising, ranging from 99.16 to 99.53. While the accuracy rates for the four studies ranged from 28 to 88. All of this resulted from using convolutional neural networks to build the model and then using PSO to improve the parameter values with the time series.

On the other hand, the proposed model was compared to the study presented by Kassaymeh^18^ using the FCNN and GWO-FC algorithms on the same datasets under the same conditions. The results showed a clear superiority of the proposed model over other models, as the error rates were almost non-existent on the MAE, MSE, RMSE, and MdMRE. In addition, the prediction accuracy results were very high. In addition, to ensure the efficiency of the proposed study, six criteria were used for comparison with previous studies, each of which used only four criteria. The proposed study was also applied to Atkinson and Telecom datasets, which were not used in previous comparative studies. The study shows distinctive and promising results for all data sets on several criteria, as shown in Table 5.

**Figure 7:**
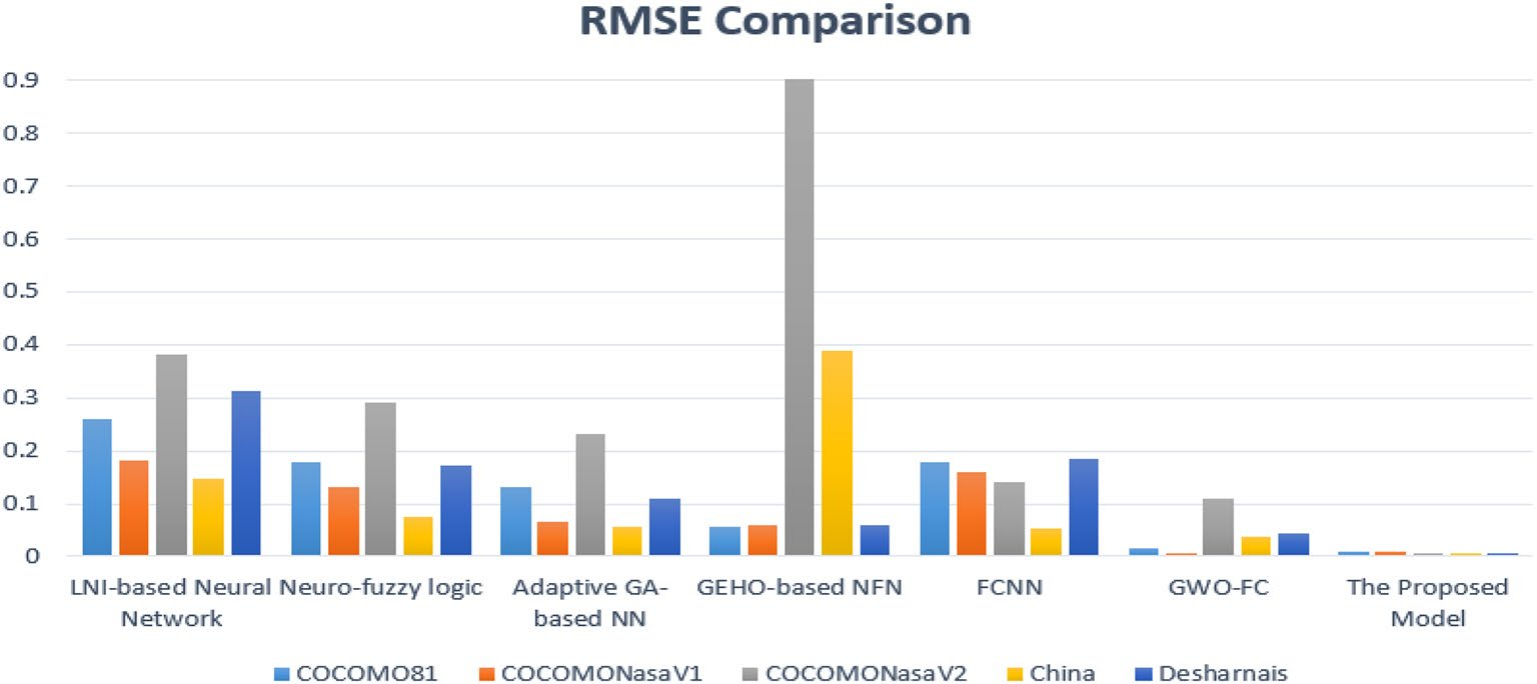
Comparison between the proposed model and others on the RSME measure.

Figure 7 shows a comparison of the COCOMO81, COCOMONasaV1, COCOMONasaV2, China, and Desharnais datasets using the RMSE measure. The results show the superiority of the proposed algorithm compared to the state of the art. The error rate decreased by percentages ranging between 0.004 and 008 for the five datasets on the proposed model, while the rate for the other algorithms were between 0.006 and 0.9. This difference shows a very high efficiency and promising results for the proposed model.

**Figure 8:**
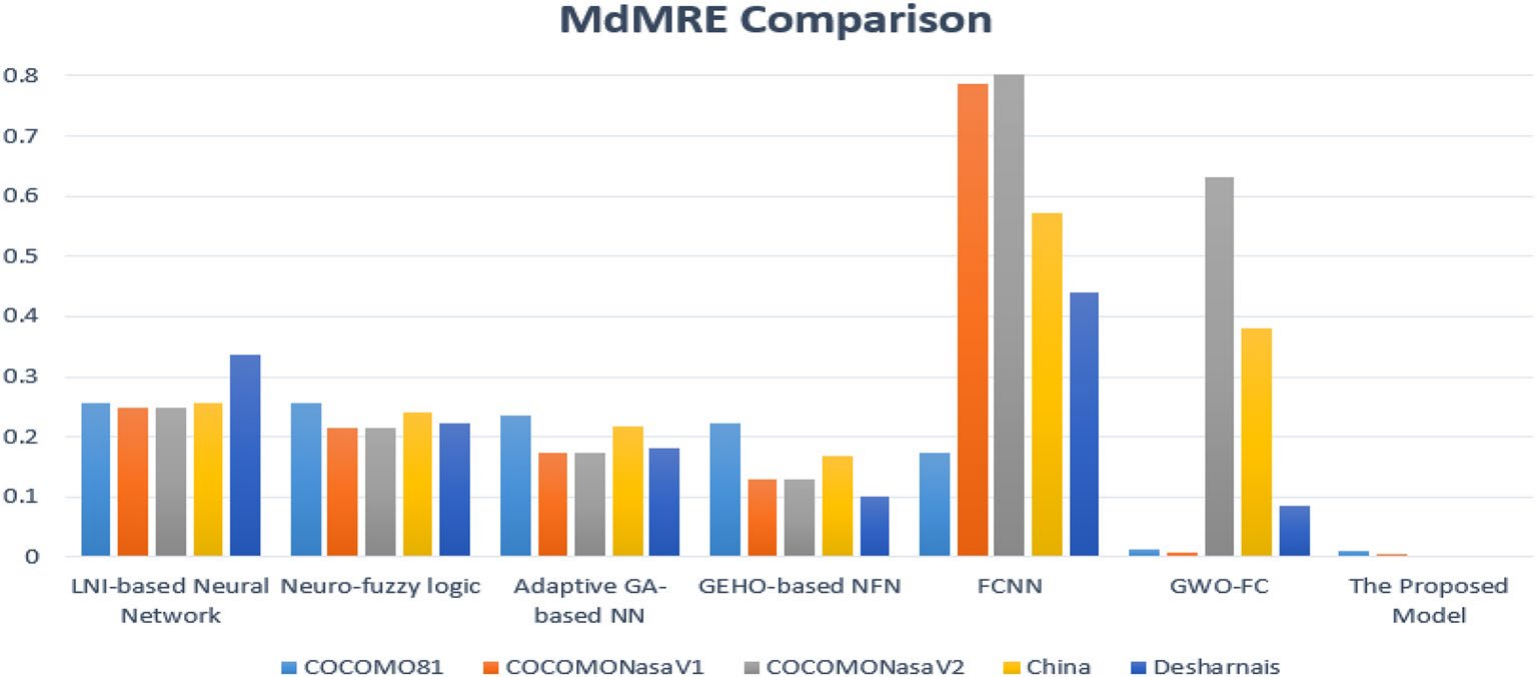
Comparison between the proposed model and others on the MdMRE measure.

The proposed model also demonstrates promising results in predicting software cost estimation according to the MdMRE scale. The results showed that the error rates are very small for the five datasets, it reaching 3.464e-05 in the Desharnais dataset. The percentages were also promising for the rest of the datasets compared to previous studies, as shown in Fig. 8.

## Conclusion

Today’s information technology is different in terms of speed and ease of business. Therefore, many institutions and companies have turned to building websites and mobile applications to keep pace with the rapid development and facilitate their work. This put great pressure on software companies, which in turn forced them to produce software at exorbitant costs. Therefore, there must be models that can predict the cost of software and the effort required to implement it. This process is known as software costing, and its importance lies in its ability to plan projects well and manage them effectively by setting goals and delivery dates. It also contributes to budgeting and financial control, allowing organizations to create budgets and allocate funds appropriately. It also shares insight into the expected costs of constructing these projects and allows for risk management by identifying and evaluating potential risks by considering several factors such as complexity, technology used, and experience of the work team. Therefore, researchers conducted many studies to predict cost estimation, although the results were unstable and inaccurate to improve the accuracy of prediction. Therefore, the proposed model sought to use machine and deep learning techniques by building a model based on the use of data science techniques, where the data was represented, processed, and visualized on Google Colab to understand it and remove noise, and then analyze the data and divide it into training and test sets. The proposed methodology used time series technology to correlate and concatenate features, convolutional neural networks were applied to extract features, and then a practical swarm optimization algorithm was applied to optimize the parameters and find the optimal solution. The proposed model was trained on thirteen datasets collected from Promise and GitHub repositories. To measure the efficiency and accuracy of the proposed model, six metrics were used: MAE, MSE, MMRE, RMSE, MdMRE, and PRED. The proposed model contributes to improving software cost estimation prediction. It also contributes to choosing the best standards that contribute to estimating the cost, as well as reducing the risks that software houses face while building their projects. It will also reduce complexity during the construction process because of its clear vision.

While the integration of CNN architecture with PSO for software cost estimation represents a promising approach, several limitations need to be addressed along with avenues for future exploration, especially with regard to real-time application. Challenges include dataset stationary assumptions, computational overhead, and fixed evaluation metrics. Future research could focus on adaptive modeling techniques, lightweight architecture, and real-time data integration to enhance model responsiveness. Leveraging online learning and reinforcement learning methods can improve the ability to adapt to evolving project dynamics. Addressing these limitations and developing real-time application domains is critical to the practical utility of CNN-PSO models in dynamic software development environments.

Therefore, in future work, we aim to apply the model in real-time in addition to building a tool with a simple user interface that enables the user to enter the software project requirements for cost calculation in a simplified manner on the proposed model.

## Data Availability

The datasets used and/or analysed during the current study are available in the Promise and GitHub repositories, [http://promise.site.uottawa.ca/SERepository/datasets-page.html, https://github.com/danrodgar/DASE/tree/master/datasets/effortEstimation].
